# Increased Breastfeeding Proportion Is Associated with Improved Gross Motor Skills at 3–5 Years of Age: A Pilot Study

**DOI:** 10.3390/nu14112215

**Published:** 2022-05-26

**Authors:** Erica E. D’Souza, Rutvi Vyas, Michaela Sisitsky, Henry A. Feldman, Borjan Gagoski, Jonathan Litt, Ryan J. Larsen, Matthew J. Kuchan, John B. Lasekan, Brad P. Sutton, Patricia Ellen Grant, Yangming Ou, Sarah U. Morton

**Affiliations:** 1 Division of Newborn Medicine, Boston Children’s Hospital, Boston, MA 02115, USA; edsouza2@student.nymc.edu (E.E.D.); rutvi.vyas@childrens.harvard.edu (R.V.); henry.feldman@childrens.harvard.edu (H.A.F.); ellen.grant@childrens.harvard.edu (P.E.G.); yangming.ou@childrens.harvard.edu (Y.O.); 2 Fetal-Neonatal Neuroimaging and Developmental Science Center, Boston Children’s Hospital, Boston, MA 02115, USA; michaela.sisitsky@childrens.harvard.edu (M.S.); borjan.gagoski@childrens.harvard.edu (B.G.); 3 Department of Pediatrics, Harvard Medical School, Boston, MA 02115, USA; 4 Institutional Centers for Clinical and Translational Research, Boston Children’s Hospital, Boston, MA 02115, USA; 5 Department of Radiology, Boston Children’s Hospital, Boston, MA 02115, USA; 6 Department of Neonatology, Beth Israel Deaconess Medical Center, Boston, MA 02115, USA; jlitt@bidmc.harvard.edu; 7 Beckman Institute, University of Illinois at Urbana-Champaign, Urbana, IL 61801, USA; larsen@illinois.edu (R.J.L.); bsutton@illinois.edu (B.P.S.); 8 Abbott Laboratories, Columbus, OH 43219, USA; matthew.kuchan@abbott.com (M.J.K.); john.lasekan@abbott.com (J.B.L.); 9 Department of Bioengineering, University of Illinois at Urbana-Champaign, Urbana, IL 61801, USA

**Keywords:** breastfeeding, neurodevelopmental outcomes, maternal nutrition, α-tocopherol, zeaxanthin, lutein

## Abstract

Breastmilk provides key nutrients and bio-active factors that contribute to infant neurodevelopment. Optimizing maternal nutrition could provide further benefit to psychomotor outcomes. Our observational cohort pilot study aims to determine if breastfeeding extent and breastmilk nutrients correlate with psychomotor outcomes at school age. The breastfeeding proportion at 3 months of age and neurodevelopmental outcomes at 3–5 years of age were recorded for 33 typically developing newborns born after uncomplicated pregnancies. The association between categorical breastfeeding proportion and neurodevelopmental outcome scores was determined for the cohort using a Spearman correlation with and without the inclusion of parental factors. Vitamin E and carotenoid levels were determined in breastmilk samples from 14 of the mothers. After the inclusion of parental education and income as covariates, motor skill scores positively correlated with breastmilk contents of α-tocopherol (Spearman coefficient 0.88, *p*-value = 0.02), translutein (0.98, *p*-value = 0.0007), total lutein (0.92, *p*-value = 0.01), and zeaxanthin (0.93, *p*-value = 0.0068). Problem solving skills negatively correlated with the levels of the RSR enantiomer of α-tocopherol (−0.86, *p*-value = 0.03). Overall, higher exposure to breastfeeding was associated with improved gross motor and problem-solving skills at 3–5 years of age. The potential of α-tocopherol, lutein, and zeaxanthin intake to provide neurodevelopmental benefit is worthy of further investigation.

## 1. Introduction

Appropriate nutrition is essential for the neurodevelopment and growth of infants [1]. Nutrition in the first months of life is typically derived from maternal breastmilk, infant formula, or a combination of the two. Breastmilk contains a unique mixture of proteins, vitamins, and other bio-active factors that help support ex utero brain development [2,3]. Infant formulas generally contain a similar nutritional profile; however, they do not contain all the physiological factors and nutrients found in human milk. Cross-sectional investigations comparing the effects of breastfeeding with formula feeding have demonstrated that breastfed infants at 2 months of age have larger whole brain, cortical, and white matter volumes [4,5]. White matter contains many myelinated axons, and the increased myelination in breastfed children is accompanied by improved cognitive and verbal abilities, whereas deficiencies in myelination have proven to be responsible for neurodevelopmental disorders persisting into childhood [6]. Developing a better understanding of the beneficial effects of breastfeeding can lead to improvement in maternal diet recommendations or formula compositions that can optimize infant health and long-term outcomes.

Although the positive association between breastmilk and early neurodevelopment is widely reported, few studies have followed the infants through till childhood to determine the longer-term effects on psychomotor development. Most studies reporting school-aged outcomes have not reported significant effects of breastfeeding after accounting for confounding variables such as maternal demographics and education [7]. Previous studies that have accounted for those confounding variables were limited by their retrospective collection of breastfeeding data [8]. Concentrations of nutrients and bioactive factors within breastmilk vary widely among women, although an exact reason has not yet been concluded. Potential explanations include that the short-term neurodevelopmental impacts do not translate to long-term changes in outcomes, a larger-magnitude influence of post-infancy factors makes the impact of breastfeeding difficult to detect, or an inaccurate breastfeeding history is ascertained due to the long time between exposure and measurement at school age.

To explore the discrepancy between early and late effects of breastfeeding on neurodevelopment, we obtained school-aged outcomes for 33 term-born infants whose mothers completed an infant intake diary at one-month and provided breastmilk samples during infancy [9]. Although the cohort for our pilot study was small, it collected unique data types, such as breastmilk carotenoid contents, that had not been explored in past studies of breastfeeding and neurocognitive outcomes. First, we assessed the relationship between breastfeeding levels and school-aged neurodevelopmental outcomes while accounting for potential confounders such as maternal age, education, and reading levels. Second, we investigated whether the nutrient composition of the mothers’ breastmilk samples may potentially mediate the relationships between breastfeeding level and neurodevelopmental outcomes. This was done by identifying the nutrients correlated with neurodevelopmental outcomes and testing whether adjustment for such nutrients could abolish the breastfeeding–outcome correlation.

## 2. Materials and Methods

### 2.1. Enrollment

**Figure A1:**
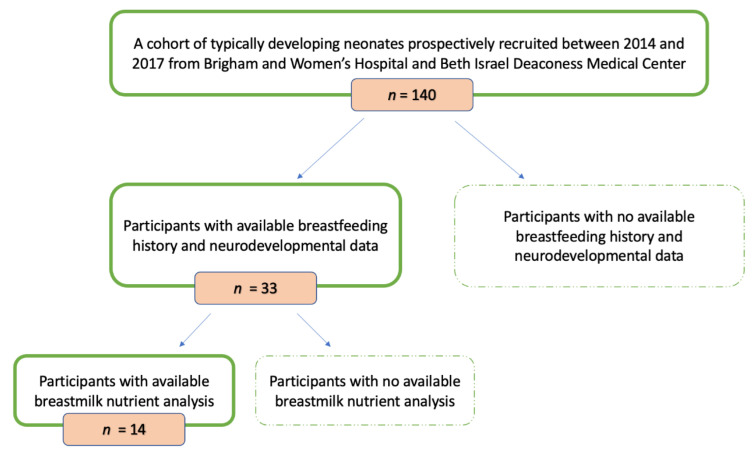
Participant flow chart from parent study cohort to subset of participants with relevant data for the current analysis.

A cohort of 161 typically-developing late-preterm and full-term neonates, whose mothers had no known medical conditions or complications during pregnancy, was prospectively recruited from Brigham and Women’s Hospital (Boston, MA, USA) and Beth Israel Deaconess Medical Center (Boston, MA, USA) between 2014 and 2017 (Appendix A Figure A1) [9]. The protocol was approved by the institutional review board at Boston Children’s Hospital and registered at clinicaltrials.gov (NCT02058225). Written informed consent of the parent or legal guardian was obtained. Patient-level data sharing was approved by the Institutional Review Board at Boston Children’s Hospital (IRB-P00008442), and all data are included as supplemental information.

### 2.2. Breastfeeding Category Determination

As part of the parent study, questionnaires were distributed to participants’ mothers when their infant was 3 months old and 4 months old. Mothers selected the proportion of feedings in the past month that were breastfeeding in the following categories according to study instructions: 0–24%, 25–49%, 50–74%, 75–94%, and 95–100%. Four infants did not have data collected at 3 months; therefore, the reported range at the 4-month data was used for the analysis for those participants.

### 2.3. Breastmilk Nutrient Analysis

Breastmilk samples were collected by the mothers using a breast pump provided by the study, either at home or during a study visit. After the first 5 mL of breastmilk were released, the subsequent 10 mL were collected. If collected at home, the breastmilk was frozen at −20 °C until study visit. After being received by the study team, the breastmilk was frozen at −80 °C until analysis. Tocopherol analysis and concentrations of lutein and zeaxanthin were obtained using high-performance liquid chromatography (carotenoids: Agilent 1290 series ultra high performance liquid chromatography autosampler pump and diode array detector (Agilent Technologies, Inc., Santa Clara, CA, USA); YMC American C30 analytical column (YMC America, Inc., Devens, MA, USA); total tocopherols: Agilent 1100 series high performance liquid chromatography autosampler and binary pump (Agilent Technologies, Inc., Santa Clara, CA, USA; Thermo Scientific BETASIL Silica-100 analytical column (Thermo Fisher Scientific, Waltham, MA, USA); Shimadzu RF-10AXL fluorescence detector (Shimadzu, Columbia, MD, USA); chiral tocopherols: Waters Acquity H class ultra performance liquid chromatography pump and autosampler (Waters, Milford, MA, USA); Diacel Chemical Industries Chiracel OD-H analytical column, (Daicel, Sakai, Osaka, Japan); Shimadzu RF-20AXS fluorescence detector (Shimadzu, Columbia, MD, USA)) [10,11]. For enantiomers of α-tocopherol, the proportion of the enantiomer with regard to total α-tocopherol was calculated. Fatty acid analysis was performed by capillary gas chromatography (Agilent 7673A Automatic Sampler (Agilent, Santa Clara, CA, USA); Agilent 6890 series Gas Chromatography (Agilent, Santa Clara, CA, USA); Supelco 0.4 mm internal diameter injection liner (Sigma Aldrich, St. Louis, MO, USA); Supelco TM-2560 capillary gas liquid chromatography column (Sigma Aldrich, St. Louis, MO, USA)) according to published standards [12]. Of the 33 participants in our cohort (see below), breastmilk samples for 14 of the mothers were analyzed.

### 2.4. Neurodevelopmental Questionnaires

Thirty-three of the 161 participant parents completed the Ages and Stages Questionnaire (ASQ), the Parent’s Evaluation of Developmental Status (PEDS), and the Child Development Inventory (CDI) neurodevelopmental questionnaires [13,14,15]. The ages of the children at the time of the questionnaire ranged from 3 to 5 years. ASQ and CDI contain approximately 50 yes/no questions relating to the developmental categories. The child receives a score based on how many milestones they have achieved; therefore, a higher score correlates with better neurodevelopmental outcomes. However, both questionnaires also contain a few questions relating to parental concerns, and a lower score would be optimal for those respective sections.

Scores were calculated per survey instructions. ASQ scores are calculated in 5 domains: communication, gross motor, fine motor, problem solving, and personal/social. CDI scores are calculated in 8 domains: social, self-help, gross motor, fine motor, expressive language, language comprehension, letters, numbers, and general development. Summary scores of the number of questions with scores <25, the number of domains with scores <25, and total number of concerns are also calculated. The PEDS questionnaire similarly contains yes/no questions for 9 developmental domains: global cognition, expressive language, receptive language, fine motor, gross motor, behavior, social/emotional, self-help, and school. However, unlike ASQ and CDI, all categories are parental concerns. Summary PEDS scores include total predictive scores highlighting areas of concern, and total non-predictive scores reflecting areas without concern.

### 2.5. Statistical Approaches

The associations between categorical breastfeeding proportion and parental demographics or neurodevelopmental questionnaire scores were determined using a Spearman correlation using SAS Studio (version 3.8, SAS Institute Inc., Cary, NC, USA). In a partially adjusted model, the age of the child during the questionnaire, the maternal age at birth, the highest level of maternal education, the highest level of paternal education, and categorized family income at birth were included as covariates. A fully adjusted model further included self-reported maternal reading level (reading speed, frequency, and perception of level relative to peers) 3 months postpartum. Spearman correlations were calculated between breastmilk nutrient contents and maternal demographics. Finally, the correlation calculations were repeated to determine the relationship between breastmilk nutrients and neurodevelopmental questionnaire scores that had previously been identified as significantly correlated with breastfeeding proportion. Significant correlations were those with *p* < 0.05 without correction for multiple comparisons.

## 3. Results

### 3.1. Patient Characteristics

Among the 33 participants with infant dietary records and school age neurodevelopmental questionnaire scores, the average gestational age was 39.6 weeks (Table 1). Two (6.1%) never breastfed and solely received formula, 6 (18.2%) were breastfed <25% of the time, 4 (12.1%) were breastfed 75–94% of the time, and the remaining 21 (63.6%) were breastfed >94% of the time. When separated by the breastfeeding category, there was a significant difference in birth weight and maternal education (Table 2). A high proportion of breastfeeding was more likely among mothers who identified as White and less likely among mothers who identified as Asian.

### 3.2. Breastfeeding Category Associated with Problem Solving and Motor Skills

Overall, the majority of participants did not have developmental concerns identified by ASQ or PEDS (Appendix A Table A1). First, the relationship between breastfeeding and overall neurodevelopment as measured by these questionnaires was examined. The total number of predictive areas in PEDS, the number of areas with scores <25 on CDI, or the total number of parental concerns on ASQ were used as summary scores. Breastfeeding category not correlated with these composite scores (Appendix A Table A2). Next, the correlation of breastfeeding with individual areas of the questionnaires were determined (Appendix A Table A3, Table A4 and Table A5). There were no significant correlations in the unadjusted model. After partial adjustment, the breastfeeding category was correlated with problem solving scores on the ASQ, and gross motor scores on the CDI (Table 3). After inclusion of maternal reading level as a covariate, breastfeeding category positively correlated with gross motor skills from ASQ and CDI.

### 3.3. Breastmilk Nutrients Correlate with Gross Motor and Problem-Solving Skills

Carotenoid and folate contents of breastmilk varied between mothers (Appendix A Table A6). To gain a better understanding of potential nutritional mechanisms for the correlation of breastfeeding category and neurodevelopmental scores, we determined whether breastmilk nutrients correlated with the neurodevelopmental scores previously identified. Problem solving skills were negatively correlated with the proportion of RSR-α tocopherol with a coefficient of −0.86 (*p*-value = 0.03, Table 4). Gross motor skills were found to be positively correlated with levels of total α-tocopherol (0.88, *p*-value = 0.02), translutein (0.98, *p*-value = 0.0007), lutein (0.92, *p*-value = 0.01), and zeaxanthin (0.93, *p*-value = 0.0068). To examine whether breastmilk nutrients could mediate the relationship between breastfeeding category and outcomes, the 5 correlated nutrient–outcome pairs from Table 4 were used to re-assess the correlation of breastfeeding category with the 3 outcomes (ASQ: gross motor, ASQ: problem solving, and CDI: gross motor), while now including the breastmilk nutrients (total α-tocopherol for ASQ: gross motor; RSR-α-tocopherol with ASQ: problem solving; and translutein, total lutein, and zeaxanthin with CDI: gross motor) in the model. After inclusion of RSR-α-tocopherol, the correlation between breastfeeding category and ASQ: problem solving became no longer significant (0.63, *p* = 0.25), consistent with RSR-α-tocopherol mediating the correlation.

## 4. Discussion

Early life experiences and exposures have lasting implications for health and outcomes. Many previous studies have well-established short-term health and developmental benefits to breastfeeding. In this analysis, we observed that higher breastfeeding proportions are associated with improved problem solving and gross motor skills at school age. Our findings are consistent with prior observations that breastfeeding positively impacts early child cognition [16,17,18,19]. Strengths of this analysis include the ascertainment of parental education as important covariates, as well as the prospective collection of breastfeeding exposure during infancy. It is important to note that the relationship between breastfeeding levels and the participants’ problem solving and gross motor skills was strengthened after the inclusion of maternal reading level as a covariate. Parental reading levels have been linked to parental cognitive stimulation, or the extent to which parents engage and enrich their child’s cognitive development [20,21]. Therefore, parental reading levels have been shown to influence child cognition by directly shaping the home environment the child grows up in, linking it to what research has described as one of the most important factors contributing to a child’s neurodevelopment and educational success [22,23].

Further, we found that breastmilk total α-tocopherol was positively correlated with gross motor scores, whereas the proportion of the synthetic RSR-α-tocopherol isomer was negatively correlated with problem solving skills. α-Tocopherol is the most biologically active form of vitamin E, an essential nutrient that is consumed through foods such as nuts, oils, and leafy green vegetables [24]. α-Tocopherol exists in nature only as RRR-α-tocopherol; however, synthetic stereoisomers, such as RSR-α-tocopherol, are present in many foods and supplements and can accumulate in the liver and brain [10,25]. However, it is not currently known whether there is an advantage of increased RRR-α-tocopherol, or if an accumulation of synthetic stereoisomers is unfavorable during brain development [26].

Because humans cannot synthesize α-tocopherol, infants acquire it from either breastmilk or formula, both of which generally contain both natural and synthetic stereoisomers of α-tocopherol. Breastmilk α-tocopherol concentrations and stereoisomer profiles are influenced by maternal diet and supplementation and may also be influenced by maternal age and gestational age [27]. α-Tocopherol acts as a lipid soluble antioxidant and helps protect membrane polyunsaturated fatty acids from free radical damage [24,27]. Recently, studies have demonstrated that α-tocopherol may prevent adverse neonatal outcomes via its antioxidant properties [28]. Supplementation with α-tocopherol reverses neurological symptoms characteristic of deficiency in humans [29,30,31]. In addition, mouse studies have demonstrated that α-tocopherol is necessary for the development of Purkinje cells in the cerebellum, and therefore vitamin E deficiency could impair motor skills [32]. However, the role of α-tocopherol on gross motor skills during early stages of development has not been well explored, making our study the first to provide evidence supporting a causal relationship between those variables. This potential mediation was supported by the relationship between breastfeeding category and ASQ, with problem solving becoming insignificant once RSR-α-tocopherol was included in the model.

In this cohort, lutein, translutein, and zeaxanthin were associated with higher gross motor scores. Lutein and its isomer zeaxanthin are carotenoids, pigments that are naturally present in plants and green, leafy vegetables [33]. Lutein is the dominant carotenoid found in adult and infant brains, particularly in the neocortex [34]. Lutein and zeaxanthin are highly concentrated in the macula, a structure with a particularly high concentration of cones unique to humans and other primates. Lutein and zeaxanthin form the yellow macular pigment. Macular pigment optical density, a correlate of brain lutein, has been found to have a positive association with cognitive abilities in the pediatric population [35,36]. Therefore, a potential link between these two carotenoids and early brain development has been hypothesized. Recently, a higher intake of lutein and zeaxanthin during pregnancy was found to be positively associated with verbal intelligence and behavior regulation in the prospective cohort Project Viva [37]. In addition, research has shown that lutein and zeaxanthin, among other carotenoids, can improve visual processing speed [38,39]. Our pilot study contributes to the growing body of evidence suggesting that lutein and zeaxanthin play an important role in neurodevelopment, and presents a new finding that gross motor skills, in particular, may be the target.

The modest sample size of our pilot study resulted in limited statistical power in some of our analyses. However, our analysis was completed on a unique data set with the availability of breastmilk carotenoid contents and was notable for inclusion of maternal reading comfort as a confounding variable that is not often included in studies of breastfeeding and neurocognitive outcomes. In addition, the generalizability of our finds may be limited by the relatively uniform educational background of our study population. Therefore, our findings need to be replicated in a larger, more diverse sample population. Spearman correlations were used in our analysis, but future studies in larger cohorts could use linear models to determine if parental factors jointly contribute to neurodevelopmental values. Finally, it is important to note that there may be other confounding factors at play. There might be variations in the results due to the parents completing the neurodevelopmental questionnaires themselves. In addition, past studies have hypothesized that the cognitive benefits of breastmilk may not be due to the nutrients in the breastmilk, but rather the skin-to-skin contact between the mother and infant during the act of breastfeeding [17].

In conclusion, children exposed to a higher proportion of breastfeeding demonstrated improved motor and problem-solving skills at school age. Breastmilk contents of α-tocopherol and carotenoids were positively correlated with motor skills, indicating that optimizing maternal diet could improve the neurodevelopment of offspring.

## Figures and Tables

**Table 1:** Cohort characteristics.

Participant Characteristics (*n* = 33)	Mean ± SD or *n* (%)
**Female**	11 (33.3)
**Gestational age (weeks)**	39.6 ± 1.25
**Birth weight (kg)**	3.35 ± 0.47
**Apgar 5 score**	8.83 ± 0.38
**Mother’s age (years)**	33.45 ± 3.26
**Mother’s self-reported ethnicity**	
**Hispanic or Latino**	3 (9.1)
Non-Hispanic or Latino	30 (90.9)
**Mother’s self-reported race ^1^**	
White	25 (75.8)
Black or African American	4 (12.1)
Asian	2 (6.1)
More than one race	1 (3.0)
**Mother’s education ^2^**	
High school diploma or equivalency (GED)	2 (6.1)
Associate degree (junior college)	3 (9.1)
Bachelor’s degree	7 (21.2)
Master’s degree	11 (33.3)
Doctorate professional	7 (21.2)
Other	3 (9.1)
**Father’s education ^2^**	
High school diploma or equivalency (GED)	6 (18.2)
Associate degree (junior college)	2 (6.1)
Bachelor’s degree	12 (36.4)
Master’s degree	8 (24.2
Doctorate professional	4 (12.1)
Other	1 (3.0)
**Family income**	
Less than $25,000	2 (6.1)
$25,000–$49,000	5 (15.2)
$50,000–$74,999	3 (9.1)
$75,000–$99,999	2 (6.1)
$100,000 or greater	21 (63.5)

^1^ One mother did not provide self-described race. ^2^ No parents reported less than high school education. Abbreviations: standard deviation, SD.

**Table 2:** Maternal demographics within breastfeeding categories.

Participant Characteristics (*n* = 33)	0% BF (*n* = 2)	1–25% BF (*n* = 6)	75–94% BF (*n* = 4)	>94% BF (*n* = 21)	Spearman Coefficient (*p*-Value)
**Female**	0 (0%)	2 (33.3%)	2 (50.0%)	7 (33.3%)	0.05 (0.79)
**Gestational age (weeks)**	39.0 ± 0.6	39.6 ± 1.4	38.2 ± 0.3	39.9 ± 1.2	0.34 (0.05)
**Birth weight (kg)**	2.9 ± 0.7	3.3 ± 0.4	2.9 ± 0.2	3.5 ± 0.4	**0.35 (0.04)**
**Apgar 5 score**	9.0 ± 0	8.6 ± 1.7	8.7 ± 0.6	8.9 ± 0.3	0.32 (0.13)
**Mother’s age (years)**	28.0 ± 1.4	34.8 ± 3.1	31.3 ± 4.7	34.0 ± 2.53	0.21 (0.24)
**Mother’s ethnicity**					−0.15 (0.39)
Hispanic or Latino	0 (0%)	1 (16.7%)	1 (25.0%)	1 (4.8%%)	
Non-Hispanic or Latino	2 (100%)	5 (83.3%)	3 (75.0%)	20 (95.2%%)	
**Mother’s self-reported race**					
White	0 (0%)	3 (50.0%)	2 (50.0%)	20 (95.2%)	**0.63 (9.17 × 10^−5^)**
Black or African American	1 (50.0%)	0 (0%)	2 (50.0%)	1 (4.8%)	−0.26 (0.15)
Asian	1 (50.0%)	1 (16.7%)	0 (0%)	0 (0%)	**−0.42 (0.01)**
More than one race	0 (0%)	1 (16.7%)	0 (0%)	0 (0%)	
**Mother’s education**					**0.42 (0.02)**
High school diploma or equivalency (GED)	0 (0%)	1 (16.7%)	1 (25.0%)	0 (0%)	
Associate degree (junior college)	1 (50.0%)	1 (16.7%)	1 (25.0%)	0 (0%)	
Bachelor’s degree	0 (0%)	2 (33.3%)	0 (0%)	5 (23.8%)	
Master’s degree	1 (50.0%)	0 (0%)	0 (0%)	10 (47.6%)	
Doctorate professional	0 (0%)	1 (16.7%)	1 (25.0%)	5 (23.8%)	
Other	0 (0%)	1 (16.7%)	1 (25.0%)	1 (4.8%)	
**Father’s education**					0.30 (0.09)
High school diploma or equivalency (GED)	0 (0%)	2 (33.3%)	3 (75.0%)	2 (9.5%)	
Associate degree (junior college)	0 (0%)	0 (0%)	0 (0%)	2 (9.5%)	
Bachelor’s degree	1 (50.0%)	4 (66.7%)	0 (0%)	7 (33.3%)	
Master’s degree	1 (50.0%)	0 (0%)	0 (0%)	7 (33.3%)	
Doctorate professional	0 (0%)	0 (0%)	1 (25.0%)	3 (14.3%)	
Other	0 (0%)	0 (0%)	0 (0%)	0 (0%)	
**Family income**					0.15 (0.40)
Less than $25,000	0 (0%)	2 (33.3%)	0 (0%)	0 (0%)	
$25,000–$49,000	0 (0%)	1 (16.7%)	2 (50.0%)	2 (9.5%)	
$50,000–$74,999	0 (0%)	0 (0%)	0 (0%)	3 (14.3%)	
$75,000–$99,999	0 (0%)	0 (0%)	0 (0%)	2 (9.5%)	
$100,000 or greater	2 (100.0%)	3 (50.0%)	2 (50.0%)	14 (66.7%)	
No response	0 (0%)	0 (0%)	0 (0%)	0 (0%)	

Abbreviations: breastfeeding, BF. Bold text in Spearman coefficient column indicates significant associations with *p* < 0.05.

**Table 3:** Correlation between breastfeeding category and questionnaire score for 33 participants with breastfeeding and outcomes data.

Question	Unadjusted *	Partially Adjusted *	Fully Adjusted *
ASQ: problem solving	0.35 (0.05)	**0.39 (0.04)**	0.39 (0.05)
ASQ: gross motor	0.13 (0.46)	0.29 (0.15)	**0.41 (0.04)**
ASQ: fine motor	0.05 (0.79)	0.10 (0.62)	0.10 (0.63)
CDI: gross motor	0.34 (0.06)	**0.47 (0.01)**	**0.52 (0.008)**
CDI: fine motor	−0.04 (0.84)	0.04 (0.86)	0.05 (0.83)
PEDS: fine motor	0.13 (0.47)	0.04 (0.83)	0.03 (0.90)

* Spearman correlation of neurodevelopmental outcomes with breastfeeding category, unadjusted; partially adjusted for demographics; or further adjusted for maternal reading level. *p* tests for zero correlation. Abbreviations: Ages and Stages Questionnaire, ASQ; Child Development Inventory, CDI; Parent’s Evaluation of Developmental Status, PEDS. Bold text significant associations with *p* < 0.05.

**Table 4:** Correlation between breastmilk nutrients and significant questionnaire categories for 14 participants with breastfeeding, outcomes, and breastmilk data.

Nutrient	Question	Spearman Coefficient (*p*-Value) *
Total α-tocopherol	ASQ: Gross Motor	**0.88 (0.02)**
RSR-α-tocopherol	ASQ: Problem Solving	**−0.86 (0.03)**
Translutein	CDI: Gross Motor	**0.98 (0.0007)**
Total lutein	CDI: Gross Motor	**0.92 (0.01)**
Zeaxanthin	CDI: Gross Motor	**0.93 (0.0068)**

* Spearman correlation of neurodevelopmental outcome with breastmilk nutrient adjusted for child and maternal age, parents’ education, maternal reading levels, and family income. RSR is the specific enantiomer of tocopherol. Bold text significant associations with *p* < 0.05.

## Data Availability

Anonymized data described in the manuscript are available to qualified investigators with IRB approval.

## Appendix A

**Table A1:** Distribution of scores within questionnaires.

Questionnaire	Mean ± SD or Number Yes (%)
**ASQ**	
Communication	54.1 ± 11.0
Gross motor	54.2 ± 7.4
Fine motor	45.2 ± 15.2
Problem solving	54.5 ± 15.6
Personal/social	50.5 ± 13.3
Hears well	30 (90.1)
Speaks like other toddlers	27 (81.2)
Easily understood	32 (96.9)
Easily understood by others	30 (90.1)
Walks, runs, and climbs like others	32 (96.9)
**ASQ concerns:**	
Family history of childhood deafness	2 (6.1)
Concerns about vision	2 (6.1)
Any medical problems	5 (15.1)
Concerns about behavior	4 (12.1)
Other concerns	10 (30.3)
**PEDS (all concerns)**	
Global cognition	9 (27.3)
Expressive language	6 (18.2)
Receptive language	3 (9.1)
Fine motor	1 (3.0)
Gross motor	0 (0)
Behavior	8 (24.2)
Social/emotional	3 (9.1)
Self help	1 (3.0)
School	3 (9.1)
Other	1 (3.0)
Total predictive	0.64 ± 1.14
Total non-predictive	0.45 ± 0.94
**CDI**	
Age of child	3.9 ± 0.6
Social	36.3 ± 4.6
Self help	30.8 ± 5.8
Gross motor	25.9 ± 2.2
Fine motor	25.1 ± 3.9
Expressive language	46.7 ± 6.3
Language comprehension	46.2 ± 7.4
Letters	6.9 ± 4.2
Numbers	10.2 ± 2.5
General development	59.0 ± 8.2
**CDI concerns:**	
Number < 25	1.3 ± 1.8
Number of areas < 25	0.8 ± 1.8
Number of problems	2.4 ± 3.4

Abbreviations: standard deviation, SD; Ages and Stages Questionnaire, ASQ; Child Development Inventory, CDI.

**Table A2:** Correlation between breastfeeding category and composite questionnaire scores.

Questionnaire Overall Score	Unadjusted Spearman Coefficient (*p*-Value)	Partially Adjusted Model Spearman Coefficient (*p*-Value)	Fully Adjusted Model Spearman Coefficient (*p*-Value)
ASQ	**−0.05 (0.77)**	**−0.21 (0.29)**	**−0.23 (0.26)**
PEDS	**−0.05 (0.64)**	**0.04 (0.69)**	**0.02 (0.84)**
CDI	**−0.16 (0.31)**	**−0.04 (0.81)**	**−0.12 (0.46)**

Abbreviations: Parent’s Evaluation of Developmental Status, PEDS. Bold text significant associations with *p* < 0.05.

**Table A3:** Unadjusted correlation between breastfeeding category and questionnaire score.

Questionnaire (*n* = 33)	Spearman Coefficient (*p*-Value)
**ASQ**	
Communication	0.13 (0.45)
Gross motor	0.13 (0.46)
Fine motor	0.05 (0.79)
Problem solving	**0.35 (0.05)**
Personal/social	0.14 (0.43)
Hears well	0.21 (0.25)
Speaks like other toddlers	0.18 (0.31)
Easily understood	0.14 (0.43)
Easily understood by others	0.15 (0.39)
Walks, runs, and climbs like others	0.14 (0.43)
Family history of childhood deafness	0.19 (0.30)
Concerns about vision	−0.007 (0.97)
Any medical problems	0.13 (0.47)
Concerns about behavior	−0.07 (0.71)
Other concerns	−0.10 (0.58)
**PEDS**	
Global cognition	−0.24 (0.19)
Expressive language	0.058 (0.75)
Receptive language	−0.09 (0.62)
Fine motor	0.13 (0.47)
Gross motor	NA
Behavior	0.05 (0.79)
Social/emotional	−0.09 (0.62)
Self help	0.13 (0.47)
School	0.07 (0.69)
Other	0.13 (0.47)
Total predictive	−0.11 (0.54)
Total non-predictive	0.057 (0.75)
**CDI**	
Social	0.26 (0.15)
Self help	−0.08 (0.65)
Gross motor	0.34 (0.06)
Fine motor	−0.04 (0.84)
Expressive language	0.03 (0.87)
Language comprehension	−0.004 (0.98)
Letters	−0.28 (0.12)
Numbers	0.03 (0.89)
General development	−0.13 (0.46)
Number <25	−0.16 (0.37)
Number of areas <25	−0.20 (0.27)
Number of problems	−0.32 (0.07)

Abbreviations: not available, NA. Bold text significant associations with *p* < 0.05.

**Table A4:** Partially adjusted correlation between breastfeeding category and questionnaire score.

Questionnaire (*n* = 33)	Spearman Coefficient (*p*-Value)
**ASQ**	
Communication	−0.01 (0.95)
Gross motor	0.28 (0.15)
Fine motor	0.10 (0.62)
Problem solving	**0.39 (0.04)**
Personal/social	0.16 (0.42)
Hears well	0.20 (0.30)
Speaks like other toddlers	0.14 (0.47)
Easily understood	−0.02 (0.90)
Easily understood by others	0.03 (0.87)
Walks, runs, and climbs like others	0.04 (0.85)
Family history of childhood deafness	0.14 (0.49)
Concerns about vision	0.15 (0.43)
Any medical problems	0.18 (0.37)
Concerns about behavior	−0.02 (0.90)
Other concerns	0.05 (0.77)
**PEDS**	
Global cognition	−0.21 (0.29)
Expressive language	0.21 (0.29)
Receptive language	0.07 (0.73)
Fine motor	0.04 (0.83)
Gross motor	NA
Behavior	0.16 (0.40)
Social/emotional	0.07 (0.74)
Self help	0.04 (0.83)
School	0.14 (0.48)
Other	0.09 (0.64)
Total predictive	−0.03 (0.89)
Total non-predictive	0.17 (0.38)
**CDI**	
Social	0.10 (0.61)
Self help	−0.02 (0.89)
Gross motor	**0.47 (0.01)**
Fine motor	0.04 (0.86)
Expressive language	0.03 (0.86)
Language comprehension	−0.11 (0.58)
Letters	−0.17 (0.38)
Numbers	0.22 (0.25)
General development	−0.02 (0.91)
Number <25	−0.13 (0.51)
Number of areas <25	−0.08 (0.67)
Number of problems	−0.19 (0.34)

Bold text significant associations with *p* < 0.05.

**Table A5:** Fully adjusted correlation between breastfeeding category and questionnaire score.

Questionnaire (*n* = 33)	Spearman Coefficient (*p*-Value)
**ASQ**	
Communication	−0.02 (0.91)
Gross motor	**0.41 (0.04)**
Fine motor	0.10 (0.63)
Problem solving	**0.39 (0.05)**
Personal/social	0.15 (0.48)
Hears well	0.20 (0.33)
Speaks like other toddlers	0.20 (0.33)
Easily understood	−0.02 (0.93)
Easily understood by others	0.03 (0.88)
Walks, runs, and climbs like others	0.07 (0.74)
Family history of childhood deafness	0.19 (0.37)
Concerns about vision	0.15 (0.46)
Any medical problems	0.21 (0.30)
Concerns about behavior	−0.08 (0.70)
Other concerns	0.06 (0.78)
**PEDS**	
Global cognition	−0.23 (0.26)
Expressive language	0.20 (0.34)
Receptive language	0.05 (0.82)
Fine motor	0.03 (0.90)
Gross motor	NA
Behavior	0.13 (0.54)
Social/emotional	0.01 (0.95)
Self help	0.03 (0.90)
School	0.10 (0.65)
Other	0.15 (0.47)
Total predictive	−0.05 (0.81)
Total non-predictive	0.13 (0.52)
**CDI**	
Social	0.12 (0.57)
Self help	−0.04 (0.85)
Gross motor	**0.52 (0.0075)**
Fine motor	0.05 (0.83)
Expressive language	0.04 (0.86)
Language comprehension	−0.11 (0.61)
Letters	−0.19 (0.37)
Numbers	0.19 (0.36)
General development	−0.02 (0.91)
Number <25	−0.16 (0.45)
Number of areas <25	−0.12 (0.58)
Number of problems	−0.24 (0.24)

Bold text significant associations with *p*<0.05.

**Table A6:** Distribution of breastmilk nutrients within cohort.

Breastmilk Nutrient	Mean ± SD
Total α-tocopherol	4.13 ± 2.24
S-α-tocopherol (% of total)	0.02 ± 0.02
RSS-α-tocopherol (% of total)	0.07 ± 0.05
RRS-α-tocopherol (% of total)	0.06 ± 0.03
RRR-α-tocopherol (% of total)	0.79 ± 0.12
RSR-α-tocopherol (% of total)	0.05 ± 0.03
13 cis-lutein	8.35 ± 3.87
13′ cis-lutein	2.11 ± 1.86
Translutein	15.34 ± 10.62
Total lutein	25.81 ± 15.37
% Translutein	0.56 ± 0.10
Zeaxanthin	8.96 ± 4.38
13 cis-beta carotene	10.78 ± 6.72
Transbeta carotene	23.22 ± 10.19
Total beta carotene	33.99 ± 14.06
% Transbeta carotene	0.67 ± 0.10
Trans lycopene	1.62 ± 0.94
Tetrahydrofolate (THF)	19.70 ± 13.13
FA	27.78 ± 18.81
MTHF	22.24 ± 8.06

Abbreviations: fatty acid, FA; 5,10-methylene tetrahydrofolate, MTHF. S, RSS, RRS, RRR, RSR are enantiomers of tocopherol.
